# Predicting Throwing Performance with Force-Velocity Mechanical Properties of the Upper Limb in Experienced Handball Players

**DOI:** 10.5114/jhk/190224

**Published:** 2024-12-06

**Authors:** Qingshan Zhang, Robin Gassier, Noémie Eymard, Félicie Pommel, Philippe Berthier, Abderrahmane Rahmani, Christophe A. Hautier

**Affiliations:** 1 School of Athletic Performance, Shanghai University of Sport, Shanghai, China.; 2 Laboratoire Interuniversitaire de Biologie de la Motricité, Université de Lyon, Villeurbanne Cedex, France.; 3 Laboratoire Motricité, Interactions, Performance, Le Mans Université, Le Mans Cedex 9, France.

**Keywords:** mechanical properties, bench press throw, shoulder rotator, throwing velocity, handball

## Abstract

This study investigated the relationship between force-power-velocity (F-P-V) mechanical variables measured during the ballistic bench press throw (BPT), shoulder isokinetic rotation strength, and the throwing velocity in handball players. Twenty-seven experienced male handball players (age: 20.0 ± 3.2 yrs, body height: 180.5 ± 6.3 cm, body mass: 73.9 ± 7.9 kg) volunteered for the investigation. F-P-V mechanical variables (i.e., theoretical maximal force [F0], velocity [V0], power [P_max_]) were obtained during the single-arm BPT and an isokinetic shoulder isokinetic internal rotation test. Throwing performance was assessed for the standing and 3-step throwing velocity. Participants were divided into a “High/Fast” and a “Low/Slow” group considering their throwing performance based on a median split analysis. A strong correlation was found between V_0_ obtained from the BPT and maximal throwing velocity for standing throwing (r^2^ = 0.51, f^2^ = 1.04) and three-step throwing (r^2^ = 0.46, f^2^ = 0.85). At the same time, P_max_ obtained from the BPT had a weak association with three-step throwing performance (r^2^ = 0.18, f^2^ = 0.22). Furthermore, no significant correlation was found between all the mechanical variables obtained from the isokinetic rotation and throwing performance (all p-values > 0.05). The High/Fast group showed that only V_0_ and P_max_ obtained from the ballistic BPT had a small to moderate effect size (ES [0.06 0.23]) compared to the Low/Slow group. This finding indicates the importance of measuring the upper limb F-P-V profile obtained from the BPT in predicting throwing performance. Thus, training programs should focus on F-P-V mechanical properties to design specific training methods to optimize throwing performance in handball players.

## Introduction

Handball is an Olympic sport that requires high technical, tactical, and physical demands during competition (Vila and Ferragut, 2019; Ziv and Lidor, 2009). Overhead throwing is one of the most critical actions in handball related to competition performance, which requires players to throw as fast and accurately as possible to score a goal (Vila and Ferragut, 2019; Ziv and Lidor, 2009). It is well known that the overarm throw is a typical ballistic movement that requires the athlete to accelerate a given ball as rapidly as possible to reach the highest velocity in the shortest amount of time. The overarm throw is a complex, fast, and discrete movement divided into six phases: wind-up, stride, arm cocking, arm acceleration, arm deceleration, and follow-through (Escamilla and Andrews, 2009; Vila and Ferragut, 2019). Previous studies have well established that muscular strength and power of the lower and the upper body are critical determinants related to throwing performance in handball players (Chelly et al., 2010; Gorostiaga et al., 2005; Kyriacou-Rossi et al., 2023; Martínez-García et al., 2021). Notably, the arm acceleration phase starts approximately 180 ms before ball release, which requires players to accelerate as the distal segments (e.g., the wrist, the elbow) reach the maximal angular velocity with the lower charge during this brief period (Wagner et al., 2010). As a result, evaluating the upper limb's explosive capacity is suggested to be included in training monitoring.

The upper limb's explosive capacity, expressed by mechanical properties such as force-production capacities and power output, is critical in determining throwing performance (Debanne and Laffaye, 2011; Petruzela et al., 2023; Yildiz et al., 2006). To date, the bench press has commonly been used to evaluate the explosive ability of the upper limbs, with mechanical variables such as velocity, power, and force against different loads (Cronin et al., 2003; Langer et al., 2022). However, it is known that maximum power is produced at optimal velocity, which is half of the maximal theoretical velocity calculated from the force-velocity relationship (Hintzy et al., 2003; Rahmani et al., 2018). Unfortunately, this optimal velocity cannot be reached during a standard bench press, even at the lowest load (5–10% 1RM) (González-Badillo and Sánchez-Medina, 2010). Recently, some authors have proposed to use the linear extrapolation of the force-power-velocity (F-P-V) relationship to estimate the meaningful theoretical maximum velocity [V0], power [P_max_], and force [F0] to evaluate external mechanical effectiveness using the bench press throw (BPT) (Hintzy et al., 2003; Rahmani et al., 2018). However, no study has demonstrated the association between the F-P-V mechanical variables obtained during the BPT and throwing performance.

Meanwhile, another variable that may be critical to throwing performance could be the ability of the internal shoulder rotators to produce a high moment of force at a fast speed. The angular velocity of internal rotation could achieve more than ~1000°/s during a throwing action (Skejø et al., 2019). During this phase, internal shoulder rotators (IR) benefit from a stretch-shortening cycle in an eccentric motion followed by rapid concentric muscle contraction to throw the ball. Thus, evaluating the concentric power and torque of the internal shoulder rotators seems pertinent using isokinetic dynamometry (Yildiz et al., 2006). The interest of the isokinetic ergometer is to isolate the function of a group of muscles and measure the capacity to produce a high moment of force at different angular velocities without concern over the duration and phases of the movement in multi-joint inertial movements. Unfortunately, no consensus has been found between the shoulder isokinetic internal rotation torque and throwing velocity (Andrade et al., 2016; Bayios et al., 2001; Pontaga and Zidens, 2014). It is true that these measurements still suffer from the limit of reproducible angular velocity on the ergometer (< 500°/s), which is much lower than what is observed in a throwing motion (>1000°/s). However, as can be done in ballistic movements, it is also possible to calculate a linear isokinetic F-P-V relationship for the shoulder's internal rotator muscles and derive the leading indicators mentioned above. It could be suspected that higher velocity output might contribute to better throwing performance due to handball players' fast internal rotation during the throwing action. More importantly, assessing whether handball players with better throwing performance display the specific F-P-V relationship would inform individualized training programs.

Considering the above, the present study aimed to *i*) investigate the F-P-V mechanical properties obtained during the ballistic bench press and *ii*) examine the shoulder’s isokinetic internal rotation F-P-V relationship with the overarm throwing velocity in handball players. We hypothesized that *i*) high effectiveness in producing V_0_ and P_max_ obtained in the ballistic bench press throw could contribute to throwing performance, whereas *ii*) isokinetic F-P-V mechanical properties obtained in the shoulder’s internal rotation would be less correlated to throwing performance. Furthermore, *iii*) handball players would display a specific F-P-V relationship due to throwing performance.

## Methods

### Participants

The sample size was estimated using G*power (Brunsbuttel, Germany) according to the previous study (Chelly et al., 2010) using a correlation test, assuming that a large effect size r = 0.6, error α = 0.05, and 1-β = 0.95; thus, the sample size required at least 24 athletes. Twenty-seven young, experienced French male handball players participated in the present study (age: 20.0 ± 3.2 yrs, body height: 180.5 ± 6.3 cm, body mass: 73.9 ± 7.9 kg, training volume: 5.7 ± 2.5 h·week^−1^, training experience: 7.8 ± 2.8 yrs). All participants were right-handed without upper- and lower-limb musculoskeletal disorders in the past six months. All testing was carried out at the pre-competitive period. All the participants followed their usual training program before the experiments and did not perform intense workouts in the past 48 hours. This study was approved by the local ethics committee of the University of Lyon (protocol code: #2018-A03013-52; approval date: 17 March 2018).

### Measures

#### Ballistic Bench Press Throw (BPT)

**Figure 1:**
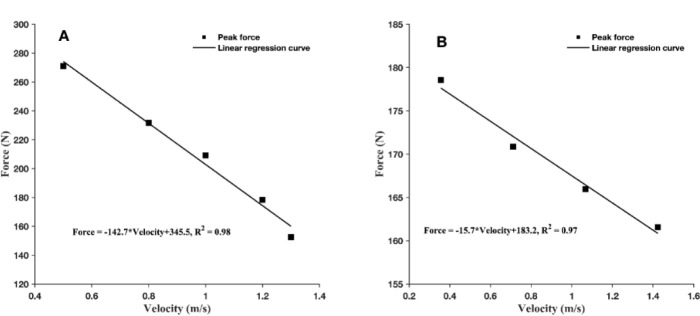
A representative set of individual peak torque (squares point) and calculation of Force-Velocity relationship curve (dashed line) based on the simple linear regression method for ballistics bench press (A) and isokinetic assessment (B), respectively.

Participants randomly performed the two trials of a single-arm BPT on a Smith machine against five different loads (10 repetitions in total) equal to approximately 15, 20, 25, 30, and 35% of body mass. The barbell was positioned across their chest at the nipple level above the pectoralis major, supported by the lower mechanical stops of the measurement device (≈ 5 cm above the chest). During the BPT, participants lay back on the bench at their non-dominant sides to hold the middle of the barbell, which permitted the measurement's reproducibility of the barbell velocity. Participants were required to push as hard and fast as possible to throw the barbell with their back entirely in contact with the bench during the push-off. Each trial was followed by a 3-minute rest interval. All the participants were asked to perform a familiarization session, including 10 single-arm BPTs, which allowed the participants to adapt to the unilateral BPT. A simple validated method was used to estimate the F-P-V mechanical properties based on three variables: the mass of the studied system (i.e., upper limbs plus lifted mass), vertical displacement during the freefall phase, and the vertical push-off distance (Rahmani et al., 2018; Samozino et al., 2008). A cable tie was fixed around the rail of the guide barbell, which could slide along the rail during the barbell lifting to measure the bar displacement. According to the previous description, the $\bar{F}$ and $\bar{V}$ were estimated as the average of instantaneous vertical force and velocity during the whole push-off phase with different charges, respectively. The F-V curve was determined by least squares linear regressions using the two trials of each of the five loads. F-V curves were then extrapolated to obtain maximal force (F_0_; force-intercept), velocity (V_0_; velocity-intercept), power (P_max_: F_0_*V_0_/4), and the linear F-V relationship (F-V slope) (Figure 1-A).

#### Isokinetic Rotation

Isokinetic measures were realized on the dominant arm, assuming the supine position with straps across the participants’ chest and hips on an isokinetic dynamometer (Contrex, 256 Hz, CMV AG, Dübendorf, Switzerland). The upper extremity was positioned with the shoulder abducted to 90° and the elbow flexed to 90°. The range of motion was fixed at 105° (i.e., 60° of internal rotation (IR) and 55° of external rotation (ER)). Participants then randomly performed five sets of three maximum repetitions of internal concentric rotation at the angular velocity of 60°·s^−1^, 90°·s^−1^, 120°·s^−1^, and 180°·s^−1^ with a 60-s rest interval between each set. Before each test, participants performed three submaximal familiarization trials with the same setup of the tests (i.e., angular velocity and ROM). The angular velocity (rad/s) and the torque (N·m) were transformed into linear velocity (m/s) and force (N), respectively, by multiplying them by the length of individual lever arms. Afterward, the F-P-V relationship of IE was assessed by fitting a linear regression through the force and angular velocity (Janicijevic et al., 2019). The F-P-V relationship was extrapolated to determine the maximum force (F_0_), maximum velocity (V_0_), maximum power (P_max_: F_0_*V_0_/4), and the slope of the relationship (F-V slope; F_0_/V_0_) (Figure 1-B).

#### Throwing Performance

Participants were instructed to perform the standing and the three-step running throw with a standard handball (480 g, circumference of 58 cm). A high-frequency sports radar was used to measure the ball velocity (100 Hz, Stalker ATS II Radar Gun, Texas, TX, USA) placed at a 3-m line behind the participants and a height of ~2 m above the ground, pointing to the executing arm (van den Tillaar, 2020). The throws were performed from the seven-meter line as the starting line in front of the cage. As for the standing throw, the participant completed the throw by holding the front foot at the starting line. Regarding the 3-step throw, players performed maximal ball throwing after a 3-step run. When throwing, players were asked to keep at least one foot before the start line. Before the test, all participants were asked to complete a familiarization session for standing and three-step running throws. To be as accurate as possible, only throws that entered directly into the goal without touching the ground in the front of the cage were considered valid. The participant was required to perform five successful trials for each throwing test, with a 40-s rest interval between each test. The best value of the throwing performances was used for further analysis.

### Design and Procedures

This cross-sectional study design investigated the association between the F-P-V mechanical variables of the ballistic bench press throw, shoulder isokinetic internal rotation, and throwing performance. Participants were required to perform two experimental testing sessions at random within seven days. During the first session, they completed two single-arm BPTs, standing, and three-step standing throwing tests. During the second session, they performed the isokinetic test.

### Statistical Analysis

The normality of the data was assessed through the Shapiro-Wilk test. Pearson’s correlation analysis with Holm correction was used to determine the relationship between throwing performance and mechanical properties. The magnitude of the correlation coefficient (r) was interpreted as very weak (0.11–0.19), weak (0.20–0.39), moderate (0.40–0.59), strong (0.60–0.79), and very strong (0.80–1.00). Additionally, players were classified as High/Fast or Low/Slow using a median split based on the better throwing performance to examine the mechanical properties of the ballistic BPT and isokinetic rotation. A linear mixed model was then used to determine the effect of throwing performance with the random intercepts as a between-subject factor on the F-P-V variables of the ballistic BPT and the isokinetic test, respectively. The partial eta-squared (η2) was used to evaluate the magnitude of differences between the groups and classified as small (0.01), medium (0.09), and large (0.25). The value of *p* was set at a 0.05 significance level. Within-test reliability was quantified using the intraclass correlation coefficient (ICC), the coefficient of variation (CV), and standard error of the measurement (SEM) (Weir, 2005). ICC values were interpreted using the following criteria: excellent (>0.9), good (0.75–0.9), moderate (0.5–0.75), and poor (<0.5). All statistical procedures were performed with R software (R 3.5.0, R Core Team, Vienna, Austria). Descriptive statistics are presented as mean ± SD with the 95% CI.

## Results

Throwing performance and mechanical variables of the F-P-V relationship obtained from the ballistic BPT and the shoulder’s isokinetic rotation are presented in Table 1. All the measurements indicated excellent reliability (ICC = 0.91–0.99) with high variability (CV = 6.92–22.18), whereas the SEM revealed a low systematic error of measurement less than 5% (Table 1).

**Table 1:** F-P-V mechanical variables obtained in the unilateral ballistic bench press and throwing performance.

	Variable	Mean ± SD	[95% CI]	ICC	CV (%)	SEM
**Throwing performance**	Standing throwing (m•s^−1^)	21.92 ± 2.25	[21.14; 22.96]	0.93	6.98	0.32
	3-steps throwing (m•s^−1^)	23.53 ± 2.26	[22.75; 24.62]	0.96	6.92	0.31
**Ballistic bench press**	F_0_ (N)	534.61 ± 135.52	[486.05; 593.18]	0.88	19.67 2	32.06
	V_0_ (m•s^−1^)	2.03 ± 0.29	[1.92; 2.16]	0.85	15.23	0.11
	P_max_ (W)	269.23 ± 75.97	[243.06; 305.64]	0.98	22.18	7.38
**Isokinetic internal rotation**	F_0_ (N)	181.62 ± 45.46	[163.26; 199.98]	0.93	10.66	3.97
	V_0_ (m•s^−1^)	7.90 ± 3.7	[6.4; 9.39]	0.98	19.01	0.13
	P_max_ (W)	351.84 ± 162.77	[286.1; 417.59]	0.97	16.68	10.31

F_0_: maximal theoretical force; V_0_: maximal theoretical velocity; P_max_: maximal power; SD: standard deviation; CI: confidence interval; ICC: intraclass correlation coefficient; SEM: standard error of the measurement

### Correlation between F-P-V Mechanical Parameters and Throwing Performance

Standing throwing velocity was positively associated with V_0_ (r^2^ = 0.51, *f^2^* = 1.04, *p*-value < 0.001) obtained from the ballistic bench press throw. Additionally, three-step throwing velocity was also correlated with V_0_ (r^2^ = 0.46, *f*^2^ = 0.85, *p*-value < 0.001) and P_max_ (r^2^ = 0.18, *f^2^* = 0.22, *p*-value = 0.03) obtained from the ballistic bench press throw. No significant association was found between F0 and throwing performance (all *p*-values > 0.05). Furthermore, no significant correlation was found between all the mechanical variables obtained from the isokinetic rotation and throwing performance (all *p*-values >0.05).

### Difference in F-P-V Mechanical Variables between High/Fast and Low/Slow Groups

**Figure 2:**
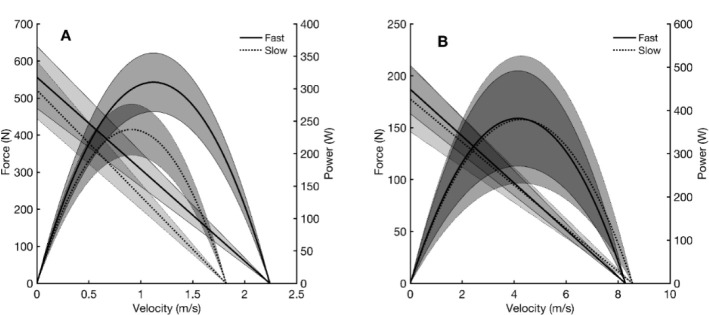
Representation of the force-velocity relationship curve from the ballistic bench press throw (A) and internal isokinetic rotation (B) displayed by throwing performance (High/Fast vs. Low/Slow); the solid and dash lines represent the curve from High/Fast and Low/Slow groups, respectively.

The High/Fast group showed higher throwing performance and mechanical variables of V_0_ and P_max_ obtained from the ballistic BPT with small to moderate effect size (0.06 < η2 < 0.23; *p* < 0.05) compared to the Low/Slow group (Table 3, Figure 2), whereas no other significant differences were founded in mechanical variables obtained from shoulder internal isokinetic rotation (Table 3, Figure 2).

**Table 2:** Associations between F-P-V mechanical variables obtained from the shoulder’s isokinetic rotation and variables of throwing.

			Correlation coefficient, r (95 CI%)	Qualitative inference	*p*-value
		Variable	(95% CI)		
V_Standing_	Bench press throw	F_0_ (N•kg^−1^)	−0.06 [-0.33; −0.43]	No effect	0.76
		V_0_ (m•s^−1^)	−0.71 [0.46;0.86]	Strong	< 0.001^*^
		P_max_ (W•kg^−1^)	−0.39 [-0.01; −0.67]	Moderate	0.04^*^
	Internal rotation	F_0_ (N•kg^−1^)	0.12 [-0.28;0.49]	Very weak	0.55
		V_0_ (m•s^−1^)	−0.09 [−0.46;0.31]	No effect	0.66
		P_max_ (W•kg^−1^)	−0.07 [−0.45;0.33]	No effect	0.73
V_Step_	Bench press throw	F_0_ (N•kg^−1^)	0.14 [−0.25;0.49]	Weak	0.48
		V_0_ (m•s^−1^)	0.7 [0.43;0.85]	Strong	< 0.001^*^
		P_max_ (W•kg^−1^)	0.44 [0.08;0.71]	Moderate	0.04^*^
	Internal rotation	F_0_ (N•kg^−1^)	0.11 [−0.29;0.48]	Weak	0.58
		V_0_ (m•s^−1^)	−0.03 [−0.41;0.36]	No effect	0.9
		P_max_ (W•kg^−1^)	−0.01 [−0.4;0.38]	No effect	0.96

F_0_: maximal theoretical force; V_0_: maximal theoretical velocity; P_max_: maximal power; F-V slope: slope of the force-velocity relationship; SD: standard deviation; CI: confidence interval

**Table 3:** F-P-V mechanical variables of the bench press throw and shoulder’s isokinetic rotation displayed by throwing performance (High/Fast vs. Low/Slow).

	High/Fast (n = 13)	Low/Slow (n = 14)	Difference		
Variable	mean ± SD	mean ± SD	Mean [95% CI]	Effect size (η2)	Qualitative inference
**Throwing performance**					
V_Standing_	23.83 ± 1.26	20.23 ± 1.69	−3.6 [−4.81; −2.38]^***^	0.276	Large
V_Step_	25.36 ± 1.27	21.9 ± 1.94	−3.46 [−4.8; −2.12]^***^	0.227	Moderate
**Bench press throw**					
F_0_	560.18 ± 150.09	521.79 ± 127.63	−38.4 [−151.33; 74.54]	0.005	Small
V_0_	2.24 ± 0.20	1.82 ± 0.22	−0.41 [−0.58; −0.24]_**_	0.203	Moderate
P_max_	309.98 ± 80.06	237.2 ± 65.14	−72.79 [−132; −13.58]_*_	0.061	Small
**External rotation**					
F_0_	147.56 ± 27.82	129.39 ± 23.41	−18.16 [−39.01; 2.68]	0.032	Small
V_0_	6.73 ± 3.94	5.87 ± 2.9	−0.85 [−3.67; 1.96]	0.004	Small
P_max_	240.01 ± 128.69	198.18 ± 117.31	−41.84 [−141.56;57.89]	0.008	Small
**Internal rotation**					
F_0_	196.73 ± 48.91	166.5 ± 37.65	−30.23 [−65.68; 5.22]	0.031	Small
V_0_	8.62 ± 4.45	7.17 ± 2.75	−1.45 [−4.48; 1.58]	0.01	Small
P_max_	403.35 ± 176.9	300.34 ± 134.75	−103.01 [−230.78; 24.76]	0.027	Small

SD: standard deviation; CI: confidence interval; V_Standing_: standing throwing velocity; V_Step_: 3-step throwing velocity; F_0_: maximal theoretical force; V_0_: maximal theoretical velocity; P_max_: maximal power; * Significantly different for p < 0.05; ** Significantly different for p < 0.01; *** Significantly different for p < 0.001

## Discussion

The present study has indicated that V_0_ obtained from the ballistic BPT is essential in determining throwing performance in handball. In contrast, none of the mechanical variables from the isokinetic shoulder rotation was related to throwing performance. Overall, throwing performances in the present study corresponded to those in amateur to elite handball, representing similar standing (21.92 ± 2.25 vs. 23.2 ± 1.6 m·s^−1^) (van den Tillaar and Ettema, 2004) and three-step throwing performance (23.53 ± 2.25 vs. 22.9 ± 1.4 m·s^−1^) (Gorostiaga et al., 2005). Furthermore, the average IR torque was similar to the broader population of handball players (Bayios et al., 2001). These similar throwing performances allow us to consider the findings within the context of handball players of the same performance level.

### Mechanical Characteristics Obtained during the BPT and Throwing Performance

As upper limb strength and power have been previously evaluated by the traditional bench press in handball players, the relative variables of the bench press (e.g., 1RM) remained challenging to predict throwing performance due to its potential deficit of expression of athletes’ mechanical properties (García-Ramos et al., 2016; Marques et al., 2007). An athlete’s capacity to accelerate and reach high throwing velocity could be partly explained by V_0_ obtained from the BPT, which reveals how effectively the upper body applies the force rapidly onto the bar. During the overhand throwing movement, the player must briefly transfer the high momentary impulse generated by proximal joints (e.g., shoulder) to the throwing arm, producing rapid, ‘whip-like’ accelerations of the arm and the hand (Roach and Lieberman, 2014). Considering that the extensor of the elbow is one of the main muscle groups engaged in the BPT, the F-P-V relationship could mainly express its contractive abilities. Thus, it could be suspected that the higher V_0_ permits the subject to reach higher velocity during the elbow extension, contributing to the higher throwing velocity during the distal acceleration phase of the throwing action (Skejø et al., 2019). As hypothesized, V_0_ was a mechanical variable the most correlated with throwing performance, which explains 51% and 52% of the variability in standing and three-step throwing velocity, respectively. This finding emphasizes the critical role of V_0_ compared to the bar velocity measured with lower loads (e.g., 20 kg) during the bench press correlated to throwing performance (coefficient correlation: ~0.71 vs. ~0.531) (Chelly et al., 2010; Debanne and Laffaye, 2011; Gorostiaga et al., 2005; Marques et al., 2007). In addition, P_max_ also correlated with throwing performance with low effect size (0.11 < r^2^ < 0.18), which is in agreement with Chelly et al. (2010) who revealed a significant association between P_max_ (r = 0.69) obtained during upper-limb cycling and throwing performance. This finding confirms again that power output of the upper limb is always crucial in determining throwing performance (Debanne and Laffaye, 2011). As a result, players in the High/Fast group presented a significantly higher V_0_ (2.24 vs. 1.92, ES = 0.23) and P_max_ (309.98 vs. 237.20, ES = 0.061) compared to the Low/Slow group (Table 3, Figure 2-A). In contrast, considering that F_0_ is the maximal capacity to produce force, it is not a surprise that there was no association between F_0_ and throwing performance because the player did not have enough time to output the maximal force during the brief acceleration phase (less than 100 ms) in the overarm throwing task (Roach and Lieberman, 2014).

### Mechanical Characteristics of Isokinetic Shoulder Rotation and Throwing Performance

Our findings agree with García-Buendía et al. (2022), who investigated the association between throwing performance and the F-P-V relationship obtained from the standing shoulder rotation. Even though the position and the method of calculating the F-P-V relationship differed, it is interesting to note that F_0_ was similar in both studies (181.44 vs. 181.62), whereas V_0_ was much higher in our study (7.90 vs. 2.97) (Garcia-Buendia et al., 2022). It could be speculated that the isokinetic shoulder rotation in the lying position allowed measuring and calculating the tangential velocity of the extremity of the segment. However, their method underestimated this velocity while calculating only the displacement of the load.

Regarding the study's second hypothesis, two possible causes could explain the absence of a significant relationship between isokinetic results and throwing performance. Firstly, the brevity of the acceleration phase and the complexity of the coordination of the different segments and joint movements (extension, flexion, rotation) during the throwing movement should be considered. This complex action poses a difficult problem for motor control, especially during the rapid throwing motion and when considering the final speed of the ball. Although the initial throwing acceleration phase recruited the shoulder’s internal rotators, V_0_ obtained from the isokinetic shoulder internal rotation in the mono-articular motion could not represent the motor control of the actual ball-throwing pattern. Secondly, the throwing action includes the stretch-shortening cycle (SSC), including an eccentric phase in the internal shoulder rotator (i.e., cocking) followed by the concentric phase (follow-through). The only shoulder isokinetic internal rotation beginning at the static position might not be an excellent movement pattern to present the dynamic handball-throwing movement. It should also be noted that the isokinetic shoulder rotation is a quasi-proximal and mono-articulation action which produces the distal extremity speed. Therefore, the proximal torque production resisting the external lever arm at a lower angular velocity could not rapidly accelerate the distal joint compared to the actual follow-through phase, which showed much higher angular acceleration and velocity. It could also be hypothesized that the internal rotation velocity measured with the isokinetic device is far below the actual velocity attained during the throwing movement. Given that the ball's mass was only ~480 g, this very light mass could not stimulate the muscle's maximal capacity for maximal power output. In brief, there was no significant difference in the mechanical variable of the isokinetic test between the High/Fast and Low/Slow groups (Table 3, Figure 2-B). Thus, the capacity to produce higher distal joint’s velocity with low loading seems more pertinent to quantify the particular explosive capacity of the internal shoulder rotators, which remains a challenge for handball players.

Meanwhile, some limitations of the present study need to be addressed. First, no kinetic and kinematic variables with EMG were measured to provide more data for current findings. Second, athletes recruited for this investigation were all male, which may limit the generalizability of the present results. Third, other variables, such as differentiating among playing positions and anthropometric characteristics, would be valuable when assessing throwing velocity and clarifying the current results. Last, the validity and reliability of force-velocity relationships measured during a single-arm ballistic BPT and isokinetic shoulder internal rotations have yet to be calculated, which should be performed in future research. However, despite greater dispersion in the measurement, the SEM and the ICC obtained in the present study indicate that these data could be used to explain handball throwing performance.

## Conclusions

The present study investigated the association between the force-power-velocity mechanical properties of the upper limb and throwing performance in handball players. The main finding indicates that V_0_ during the BPT may be key in producing high ball velocity during standing and three-step throwing. In contrast, mechanical variables derived from isokinetic shoulder rotation failed to explain throwing performance. From a practical point of view, measuring the F-V-P relationship during the ballistic BPT is necessary to demonstrate the mechanical properties of the upper limb. Training programs should focus on F-P-V profile-based training to improve specific mechanical properties through resistance training exercises and ballistic movements, such as the bench press throw and medicine ball throwing, which require the athlete to exert as much force as possible against a light load in a short time. Consequently, it seems reasonable to suggest that an individualized training program to optimize the F-P-V profile via a higher V_0_ profile may benefit handball players aiming to enhance throwing performance.
